# Preparation of Heavy Metal Trapping Flocculant Polyacrylamide–Glutathione and Its Application for Cadmium Removal from Water

**DOI:** 10.3390/polym15030500

**Published:** 2023-01-18

**Authors:** Wenjie Ding, Yunyan Wang, Weizhi Zeng, Hui Xu, Bingxin Chen

**Affiliations:** 1 School of Metallurgy and Environment, Central South University, Changsha 410083, China; 2 Chinese National Engineering Research Center for Control & Treatment of Heavy Metal Pollution, Changsha 410083, China

**Keywords:** synthesis, polyacrylamide, grafting, glutathione, flocculant, cadmium, response surface methodology

## Abstract

In this study, a heavy metal trapping gel with multiple ligand groups was prepared for the first time using response surface methodology. The gel was produced by condensing and grafting glutathione as a grafting monomer onto the main polyacrylamide chain, based on the Mannich reaction mechanism with formaldehyde. FTIR, SEM, TG-DSC, and zeta potentials were used to characterize the gel. The results demonstrated that the gel was morphologically folded and porous, with a net-like structure, which enhanced its net trapping and sweeping abilities, and that glutathione was used to provide sulfhydryl groups to boost the metal trapping ability of polyacrylamide. Coagulation experiments showed that the highest efficiency of the removal of Cd ions from water samples was achieved when the concentration of polyacrylamide–glutathione was 84.48 mgL^−1^, the concentration of Cd was 10.0 mgL^−1^, the initial turbidity was 10.40 NTU, and the initial pH was 9.0. Furthermore, the presence of two cations, Cu and Zn, had an inhibitory effect on the removal of Cd ions. In addition, analysis of the zeta potential revealed the flocculation of polyacrylamide–glutathione. The flocculation mechanism of glutathione is mainly chelation, adsorption bridging, and netting sweeping.

## 1. Introduction

As the primary source of cadmium pollution, the unregulated disposal of zinc-cadmium smelting waste and cadmium-containing battery waste may pose a substantial risk [1]. It is widely acknowledged that cadmium is a non-essential element for the human body and that excessive cadmium can cause severe damage to the human liver, kidneys, and bones and cause cancer [2]. In addition, cadmium contamination poses a significant threat to aquatic organisms and the entire ecological cycle. Therefore, cadmium management in aquatic systems is the current focus of water treatment research. As a result of its simplicity, dependability, and effectiveness, flocculation is widely used to remove heavy metals from water [3].

Traditional inorganic flocculants effectively remove colloidal substances, suspended particles, and turbidity from water, but it is more difficult to remove dissolved heavy metal ions [4]. In actual applications, the factors affecting the coagulation effect (agent dose) are complex, including pollutant type, pollutant concentration, external water conditions, and water temperature, pH, and alkalinity, among others [5,6,7,8,9,10]. Since the flocculation process of organic flocculants is a hydrolysis reaction, which requires a greater calorific value, its co-agglomeration effect (dosage) is more influenced by temperature and other variables [11]. Organic flocculants have several advantages over inorganic flocculants, including a larger floc, greater floc strength, greater mixing resistance, a lower calorific value, and no increase in sludge volume [12,13,14,15]. As a result of the ongoing research and development into organic polymer flocculants, some novel flocculant compositions have emerged [16,17,18]. Recent research has demonstrated that polymer flocculants with heavy metal retention functions can be manufactured by chemically incorporating heavy metal ions with strong coordination groups into polymer flocculants [19,20,21]. In addition to removing dissolved heavy metal ions from water via coordination or chelation, these flocculants may also effectively reduce water turbidity via electrical neutralization, adsorption bridging, and net sweeping of the floc [22]. Among the aforementioned organic polymer flocculant enhancement studies, polyacrylamide is the most studied and utilized due to its broader applicability and cheaper delivery [23,24]. Due to its reducing properties and sulfhydryl groups, glutathione is an excellent material for chelating heavy metals [25,26]. However, the current research on polyacrylamide-based flocculant grafting lacks investigation of glutathione-related effects. Thus, the authors conducted a series of experiments to investigate the potential preparation and application of this flocculant with metal chelating capability.

According to the mechanism of the Mannich reaction [27,28], the reactive hydrogen of the amide group on polyacrylamide was condensed with formaldehyde and glutathione in a subsequent study. Polyacrylamide–glutathione, a new flocculant capable of capturing heavy metals, was created by grafting sulfhydryl-containing glutathione onto polyacrylamide. It is possible to achieve both flocculation and cadmium ion removal via chelation. Response surface methodology was used to optimize the synthetic procedure, and a model was used to determine the degree of interaction between several crucial parameters. Additionally, the product was characterized to confirm the properties and morphology of the moieties. In addition, flocculation tests were conducted under various water quality conditions to evaluate the final removal of the heavy metal cadmium and determine the factors that influenced it. It is anticipated that this will serve as a significant reference for using polyacrylamide–glutathione in treating cadmium-containing wastewater.

## 2. Experiment

### 2.1. Material

The polyacrylamide (molecular weight 348 × 10^4^–521 × 10^4^) used in the experiments was synthesized directly in the laboratory using acrylamide polymerization. Acrylamide (99.5%) and reduced glutathione (99% biotech grade) used in the experiments were provided by McLean Biochemical Co. Ltd. (Shanghai, China). Ammonium persulfate, formaldehyde, manganese sulfate, potassium chloride, hydrochloric acid, sodium hydroxide, and kaolin were provided by Sinopharm Chemical Reagent Co.Ltd. (Shanghai, China). None of the purchased reagents in this experiment necessitated additional purification (except acrylamide and reduced glutathione). Deionized water was utilized for the preparation and cleaning of every solution.

### 2.2. Preparation

The target product of this study, polyacrylamide–glutathione, was synthesized in two steps: first, the amide group was reacted with formaldehyde to form N-hydroxymethyl polyacrylamide. The glutathione was then condensed and attached to N-hydroxymethyl polyacrylamide. The specific synthetic procedure required to obtain polyacrylamide-glutathione is outlined below.

#### 2.2.1. Polyacrylamide Synthesis

In a conical flask, 16 g of acrylamide was added to 80 mL of deionized water and stirred until the acrylamide was dissolved. Then, 1.5 g of carbamide dissolved in 20 mL of deionized water was thoroughly combined in the conical flask. In addition, 3 g of the initiator ammonium persulfate was added under nitrogen protection, followed by 10 min of continuous mechanical stirring. After the preceding steps were performed, the nitrogen-protected bottle was sealed and placed in a 45 °C water bath for four hours.

#### 2.2.2. Glutathione Grafting

The above-prepared sample of polyacrylamide gel (6.0 g) was completely dissolved in 100 mL of deionized water and sealed in a 200 mL three-neck flask by continuous, slow stirring for 20 min. An amount of 0.5 mL of glutathione solution at a concentration of 1 g/mL was prepared and injected into the three-necked flask, which was mechanically stirred at 30 rpm for 2.5–4 h and maintained at a constant temperature of 35 °C. After the reaction, the obtained viscous clear liquid was filtered and transformed into a white granular solid in a vacuum-dried oven at 55 °C. These pellets were identified as polyacrylamide–glutathione and ground into a powder for storage in an airtight container.

### 2.3. Characterization

On a Nicolet 6700 FT-IR spectrometer (Thermo Fisher Scientific, Waltham, MA, USA), FT-IR spectra were recorded with the measured wavenumber interval ranging from 500 cm^−1^ to 4000 cm^−1^. A JSM IT500 scanning electron microscope (JEOL Japan Electronics Co., Ltd., Beijing, China) with an accelerating voltage of 15.0 kV was utilized to observe the surface morphology. On a DTG-60H differential thermal/thermogravimetric simultaneous thermal analyzer (Shimadzu (Beijing) Co., Ltd., Beijing, China) using 40 mL/min of flowing air at a heating rate of 20 °C/min, thermal gravimetric analysis was performed. At 25 °C, the zeta potential was determined using dynamic light scattering (DLS) and a Zeta Nano ZS 90 (UK Malvern (China) Co., Ltd., Shanghai, China). In addition, cadmium and other ion concentrations were measured using inductively coupled plasma emission spectrometry (ICP, Agilent 5100, USA Agilent (China) Co. Ltd., Shanghai, China).

### 2.4. Flocculation Test

Simulated and formulated with cadmium sulfate and kaolin, the cadmium-containing water samples used in the coagulation tests were simulated to contain cadmium. Amount of 1.0 mg–20.0 mg of cadmium ions and 600 mg of kaolin were added to 1 L of deionized water, which was then mixed and stored for 30 min. Following this, 800 mL of the suspension was extracted and stored as a kaolin stock solution. The first stage was stirred at 160 r/min for 4 min, the second at 50 r/min for 10 min, and the third stage was left for 40 min. One minute after adding 200 mL of cadmium-containing simulant to the coagulation beaker, 1 to 5 mL of polyacrylamide–glutathione solution was added separately while stirring. Test samples were collected and filtered one centimeter beneath the liquid’s surface through a 5-micron filter. To measure cadmium removal in flocculation tests, the cadmium removal rate, c/c0, was calculated, where c represents the concentration of cadmium ions in real-time and c0 represents the initial concentration.

## 3. Discussion

### 3.1. Response Surface Method to Optimize the Preparation

The polyacrylamide–glutathione synthesis is influenced by a variety of external factors. Therefore, screening for these influencing factors is required to examine the relevant optimum parameters. Six single-factor experiments were conducted based on previous research on the graft modification of polyacrylamide-based flocculants [29]. However, single-factor tests are flawed in evaluating the significance of the influencing factors and the degree of interaction between them; consequently, it is frequently necessary to screen the results of single-factor tests to identify the most influential factors for the next step of experimental statistical analysis. The orthogonal test and response surface methods are the most common experimental statistical analysis techniques in materials synthesis [30]. Even though the orthogonal experiment method can identify the optimal value, the optimization region is difficult to distinguish visually and can only evaluate isolated experimental situations. However, the improved response surface method applies a polynomial model to the unknown complex function relationship within a restricted region [31,32,33]. The enhanced model is sequential and can be continuously evaluated for each level of experimental settings, resulting in more precise fitting results.

#### 3.1.1. Single-Factor Test for Synthesis Conditions

**Figure 1:**
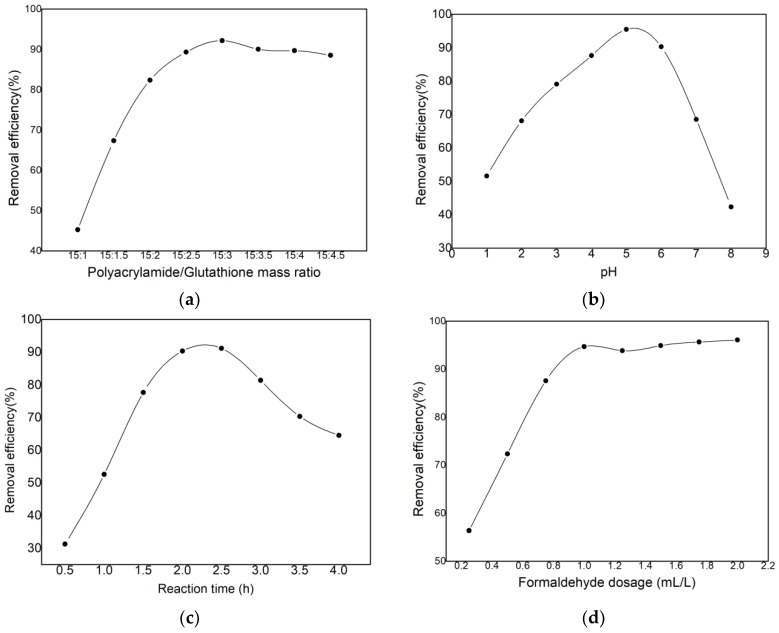

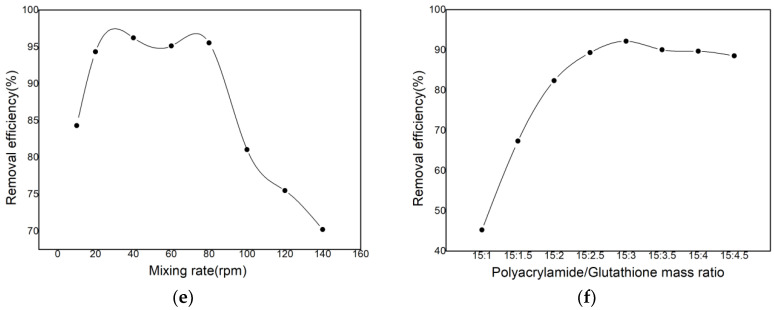
Effect of synthesis conditions on the cadmium removal rate of polyacrylamide–glutathione (**a**) m(polyacrylamide)/m(glutathione)ratio, (**b**) reaction time, (**c**) pH, (**d**) formaldehyde dosage, (**e**) mixing rate, and (**f**) temperature.

Based on the available literature and the results of the laboratory synthesis process [34,35,36,37,38,39], the following six factors were selected as significant parameters for investigating the grafting efficiency of glutathione: temperature, stirring rate, polyacrylamide/glutathione mass ratio (P/G), reaction time, initial pH, and formaldehyde concentration. Since the grafted polyacrylamide–glutathione contains sulfhydryl groups capable of chelating cadmium ions, these parameters will directly impact the cadmium ion removal efficiency of the final product. Consequently, the optimal range of the six aforementioned variables can be determined using the cadmium ion removal rate as a guide. The removal rate of cadmium ions is calculated by c/c0, where c is the concentration of cadmium ions in the sampled solution and c0 is the initial concentration of cadmium ions in the sampled solution. The initial concentration of cadmium was determined to be 15 mg L^−1^. Figure 1 depicts the experimental outcomes.

Figure 1a demonstrates that the optimal mass ratio for synthesis (m(polyacrylamide)/m(glutathione)) is 15:3. This is because the efficiency of condensation of N-hydroxymethyl polyacrylamide with glutathione does not simply increase with an increase in glutathione content, but rather there is an oxidation of -SH to form disulfide bonds (-S-S-), decreasing the amount of product carrying the ligand group -SH and forming chelates, resulting in a decrease in the removal of cadmium ions.

Figure 1b demonstrates that the ideal reaction time was 4 h. When the reaction time exceeded 4.0 h, the removal rate of Cd ions from the solution by polyacrylamide-glutathione decreased significantly, which may be due to two factors. On the one hand, the -SH was oxidized to form disulfide bonds (-S-S-) under prolonged stirring conditions. On the other hand, prolonged stirring caused a break in the molecular chain structure of polyacrylamide–glutathione, resulting in a decrease in its molecular weight [40].

Figure 1c displays the optimal pH for the reaction system as 5.0. When the pH is low, a cross-linking reaction between a high concentration of H+ and hydroxymethyl (-CH_2_OH) in N-hydroxymethyl polyacrylamide occurs [41], which is not conducive to the condensation of glutathione with N-hydroxymethyl polyacrylamide; however, the stability of glutathione decreases when the pH is greater than 5.0. According to some sources, glutathione is stable in the pH range of 2.0 to 4.0 and is easily oxidized in alkaline conditions [42].

Figure 1d demonstrates that the optimal formaldehyde concentration was 2.0 mL. Increasing the formaldehyde concentration gradually increased the production of polyacrylamide–glutathione and the elimination of cadmium ions. However, formaldehyde concentrations exceeding 2 mL result in an excess of N-hydroxymethyl polyacrylamide in the reaction system, but insufficient glutathione prevents the reaction from proceeding.

Figure 1e shows that the optimal stirring speed was 40 rpm. Increasing the speed of stirring increases the mass transfer rate of the reactants, resulting in a faster rate of polyacrylamide–glutathione synthesis. However, excessively rapid stirring increases the solubility of air in the reaction solution and oxidizes the sulfhydryl groups, thereby decreasing the chelating activity.

Figure 1f demonstrates that the optimal reaction temperature was 40 °C. The increase in temperature accelerated the mass transfer rate of the reactants, thereby increasing the rate of polyacrylamide-glutathione synthesis. On the other hand, high temperatures accelerated the oxidation of the sulfhydryl groups. Both factors decreased the rate of product synthesis.

Based on the aforementioned experiments, the following single-factor test conditions were determined to be optimal: the mass ratio of polyacrylamide to glutathione was 15:3, the glutathione grafting reaction time was two hours, the amount of formaldehyde was 1.0 mL/L, and the initial pH of the reaction system was 5.0. In addition, the obtained polyacrylamide-glutathione cadmium ion removal rate was 96.53%. Therefore, it can be said that the optimal conditions for the gel preparation have been attained.

#### 3.1.2. Experiments Design for Response Surface Optimization

Four of the most influential parameters were chosen based on their peak during the experimental period. Due to its lowest value during the interval required to remove approximately 80% of cadmium, the temperature was not significantly influencing among the parameters and was therefore not considered. While the effect of stirring speed was not considered because excessively high rotational speed is destructive to polyacrylamide-glutathione and excessively high energy consumption is impractical for the application, the effect of stirring speed was still not disregarded. In addition, screening factors with significant effects on the design of the response surface methodology were deemed effective in reducing model fitting errors and enhancing the applicability of the results. Four factors were therefore chosen for response surface analysis.

Quantifying the effects of these experimental parameters using the response surface method is necessary because the experimental and analytical results cannot be used to determine the relative importance of the experimental parameters affecting the response. The method approximates the relationship between the inputs and outputs of an entire complex system using a hypersurface. Experiments were conducted using the software-derived operating conditions, and a second-order model was fitted to the experimentally obtained data to obtain a quadratic regression equation (containing single, square, and interaction terms). The dominant and interaction effects of each effector were investigated to identify the most significant effector and the optimal solution.

Using the software’s Box–Behnken design, a 4-factor, 3-level experimental design and analysis were conducted, with the experimental factors and level values detailed in Table 1. The next step in analyzing the response surface model fitting was to enter the corresponding test results for the experimental conditions listed in Table 2. Using the data in Table 2, linear, 2FI, quadratic, and cubic prediction models were developed using the Box–Behnken method’s central composite design principle. The fit (R^2^), R^2^_adj_, and R^2^_pre_ analyses of these models are presented in Table 3.

R^2^ should be greater than 0.8 for a good-fitting model, and the closer the R^2^ value is to 1, the better. R^2^_pre_ and R^2^_adj_ values should also be close to each other to ensure the model’s predictions are close to those of the actual experiments. From Table 3, it can be seen that the fitting results of Design-Expert software recommend the use of the quadratic model (quadratic). The R^2^ value of the linear model was 0.7643, and the R^2^_adj_ and R^2^_pre_ were 0.6541 and 0.2930, and the value of the difference was less than 0.1. This indicates that the linear model has the smallest deviation and a better fit than other models. Furthermore, the final equation in terms of actual factors can also be fitted:

Removal efficiency = +66.50 + 2.41 × A − 2.47 × B + 2.63 × C + 9.13 × D + 0.23 × AB − 0.09 × AC + 0.08 × AD + 1.05 × BC + 1.19 × BD − 0.78 × CD − 0.118 × A^2^ − 0.828 × B^2^ − 0.31 × C^2^ − 2.21 × D^2^

where A represents the polyacrylamide/glutathione mass ratio; B represents time; C represents pH; and D represents formaldehyde dosage.

**Figure 2:**
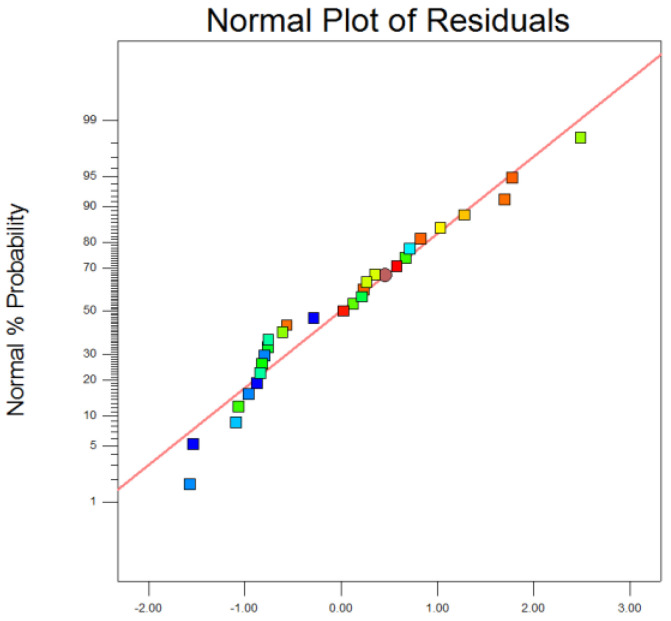
External studentized residuals.

Figure 2 depicts the distribution of the external studentized residual data points from the above quadratic polynomial regression model. The externally studentized residuals were used to determine whether the error terms follow a normal distribution [43] by indicating the degree to which the predicted values deviate from the measured values. As shown in Figure 2, the experimental points are evenly distributed, and the distribution of each point of the residuals is almost on the same line, which again demonstrates that the model’s fit is accurate and reliable.

#### 3.1.3. ANOVA and Model Significance Analysis

ANOVA was performed on the fitted model (Table 4) to determine the significance of the parameters’ influence on the response values. The greater the F-value, the greater the effect of the factor on the response value, and the smaller the *p*-value, the greater the effect of the factor on the response value. For example, the ANOVA results in Table 4 reveal that the F-value of the model was 4.78, and the *p*-values of items A, B, D, BC, and A2 in the model were <0.05, indicating that they were all significant. The order of significance of the effects of every single factor was reaction time > polyacrylamide/glutathione mass ratio > formaldehyde dosage > pH.

The model’s F-value of 8.53 indicates its significance. There is a 0.02% chance that an F-value of this magnitude could be caused by noise. Values of Prob > F of less than 0.0500 indicate significant model terms. Model terms A, B, and D are significant in this instance. Values exceeding 0.1000 indicate that the model terms are not statistically significant. The F-value for Lack of Fit of 0.94 indicates that the Lack of Fit is not significantly different from the pure.

#### 3.1.4. Response Surface Model Analysis

**Figure 3:**
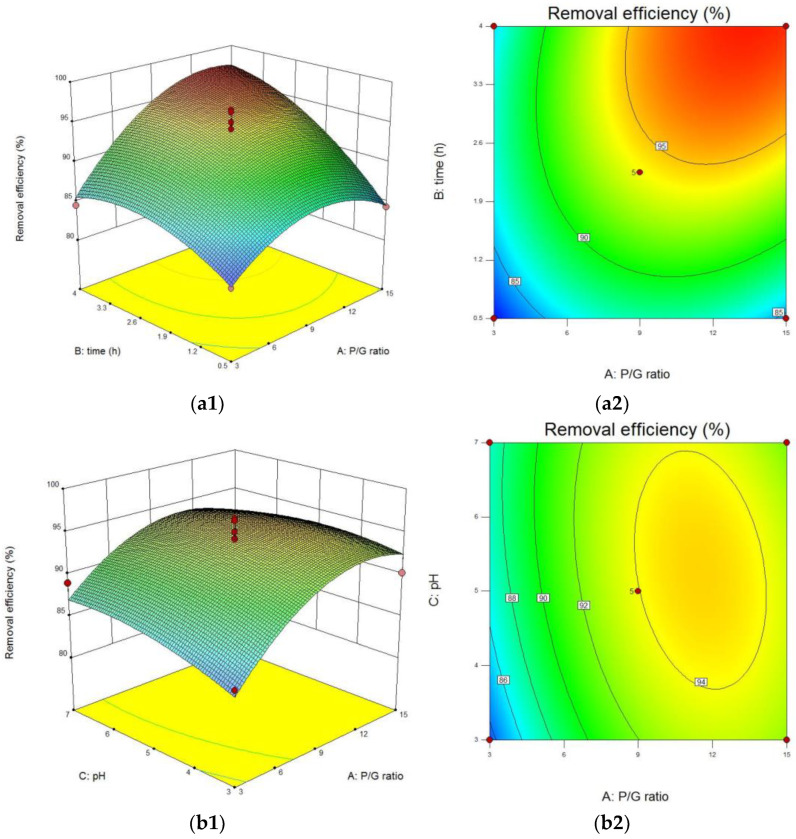

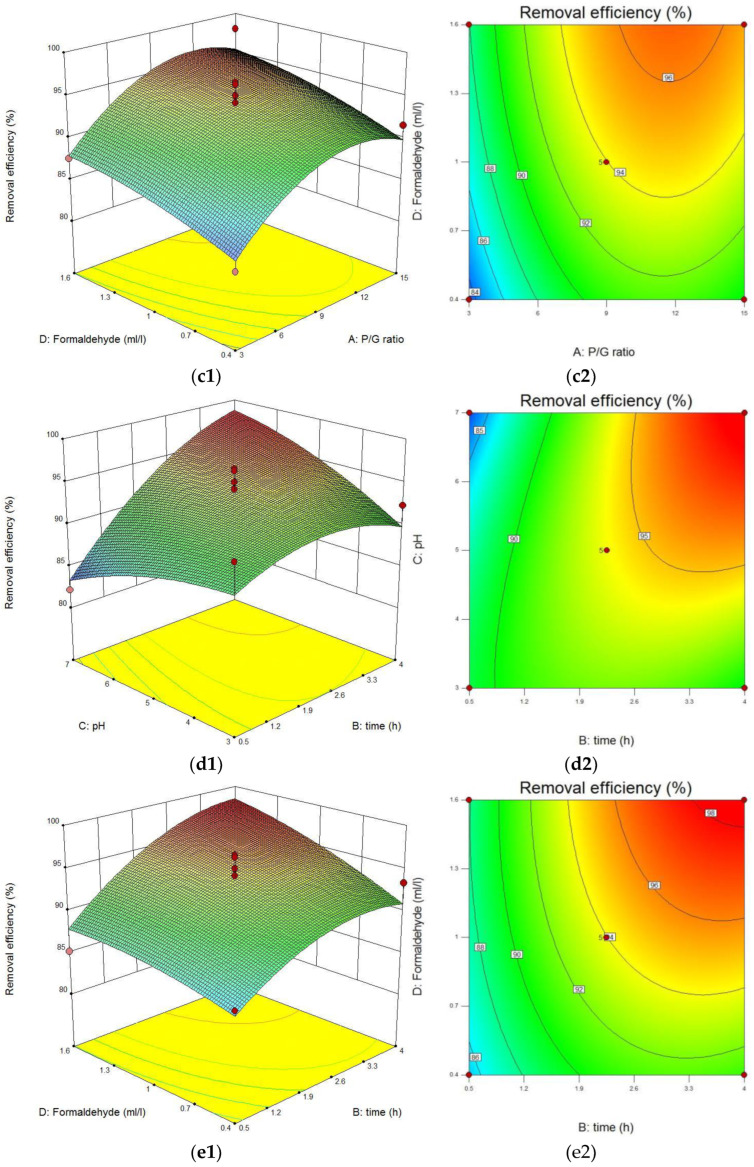

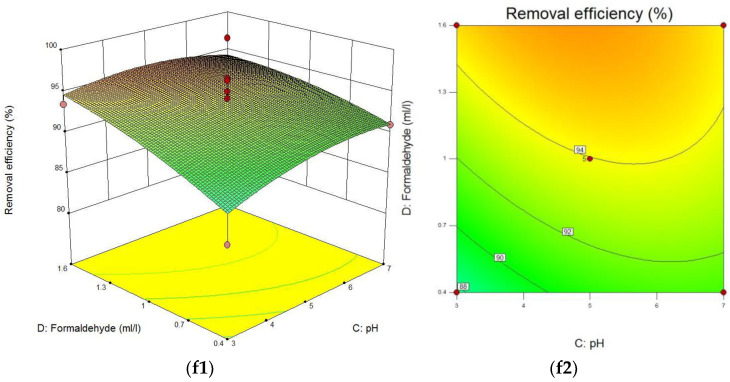
Contour map and response surface curve. (**a1**) Response surfaces of A and B and (**a2**) contour plots of A and B; (**b1**) response surfaces of A and C and (**b2**) contour plots of A and C; (**c1**) response surfaces of A and D and (**c2**) contour plots of A and D; (**d1**) response surfaces of C and B (**d2**) contour plots of C and B; (**e1**) response surfaces of B and D and (**e2**) contour plots of B and D; (**f1**) response surfaces of C and D and (**f2**) contour plots of C and D.

As shown in Figure 3, contour plots and response surface plots can be computed using the model based on the aforementioned results to create four interacting parameters that influence the cadmium removal rate. By identifying two of the factors, the effect of the remaining two factors on the response values was investigated. The steeper the slope of the response surface, the greater the effect of each factor on the response value; conversely, the opposite is true for a smaller slope.

As seen in Figure 3, the interaction between the polyacrylamide/glutathione mass ratio (A) and reaction time (B) had the greatest impact on the cadmium removal efficiency. In contrast, the interaction between formaldehyde dosage (C) and pH (D) had the least impact on cadmium removal efficiency. This result is consistent with the ANOVA results presented above. Combined with the gradient of response surface slope and contour plot variation, this response surface slope was relatively flat, and the adjacent contour interval was wide in the range of formaldehyde dose variation, indicating that the effect of formaldehyde dose on cadmium removal rate was less pronounced than that of pH and polyacrylamide/glutathione mass ratio. The significance ranking of the effect of the above four groups of parameters on cadmium removal rate was as follows: reaction time (B) > polyacrylamide/glutathione mass ratio (A) > pH (D) > formaldehyde dose (C).

#### 3.1.5. Model Optimization Results Validation

After establishing the model to further determine the optimal synthesis factors of polyacrylamide–glutathione, the DesignExpert 9.0 software was utilized to screen for parameter optimization. The results are shown in Table 5 after adjustment of the mass ratio of polyacrylamide to glutathione, the pH value of the reaction system and the dosage of formaldehyde within the reaction range and after establishment of the maximum cadmium removal efficiency as the target value. The most significant parameters were a mass ratio of 11.17:1, a reaction time of 2.692 h, a formaldehyde concentration of 0.49 mL/L, and a pH value of 4.614. When three parallel groups of experiments confirmed the model, the mean cadmium removal rate was 96.67%. With a relative error of 1.05%, the theoretically predicted value of the cadmium removal rate obtained from the regression equation was close to the actual value.

### 3.2. Characterization

In this section, a series of characterizations, primarily FTIR, SEM, and DTG, were conducted. The FTIR spectra were primarily used to compare the polyacrylamide prior to synthesis with the polyacrylamide–glutathione after synthesis and to determine if the functional group grafting was successful. EDS was used to analyze the principal elements and characterize the presence of sulfhydryl groups from an elemental perspective, while SEM was used to clarify the microstructure and morphology. Using differential thermal thermogravimetry, the thermal stability of the final reactants was evaluated. The details are listed below.

#### 3.2.1. Functional Group Analysis via FTIR

**Figure 4:**
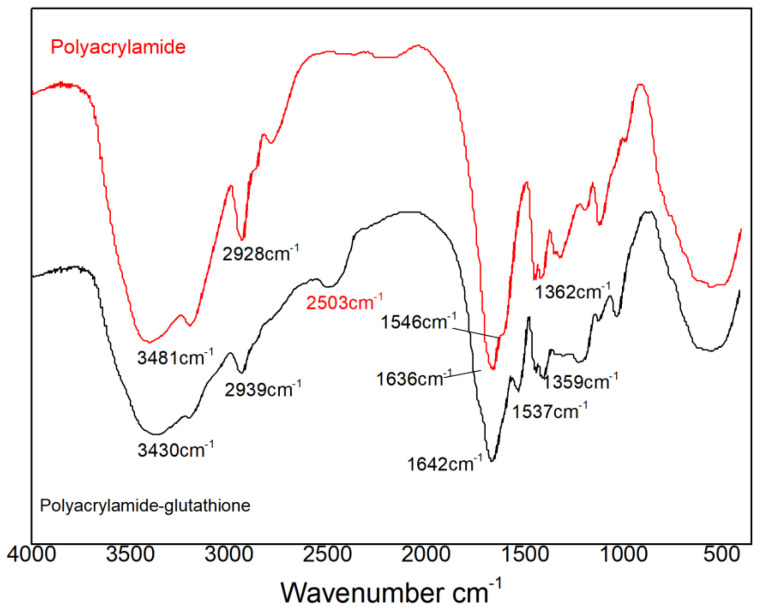
Comparison of FTIR spectra of polyacrylamide before (Red curve) and after grafting glutathione (Black curve).

Figure 4 depicts the Fourier transform infrared spectra of polyacrylamide (PAM) and polyacrylamide–glutathione (PAMG) that were separately characterized. Specifically, the characteristic peak of -CO on the amide group was observed at 1636 cm^−1^, the sharp and weak absorption peak of -COO- was observed at 1544 cm^−1^, and the characteristic peak of -CH- was observed at 1359 cm^−1^. These characteristic absorption peaks coincide with the product polyacrylamide and possess the characteristics of the final product. In the polyacrylamide–glutathione FTIR spectra, the characteristic peaks of the aforementioned polyacrylamide appeared at 3430, 2939, 1642, 1537, and 1361 cm^−1^, respectively. In particular, the characteristic peak of the target sulfhydryl group was located at 2500 cm^−1^ in the polyacrylamide–glutathione spectrum for this comparative test. Due to the weak absorption intensity of the -SH group and its susceptibility to intermolecular hydrogen bonding, the characteristic absorption peak of the -SH group depicted in Figure 4 was weak. In addition, this characteristic peak demonstrated that the synthesis experiment successfully attached glutathione to the polyacrylamide backbone.

#### 3.2.2. Surface Morphological Analysis via SEM

**Figure 5:**
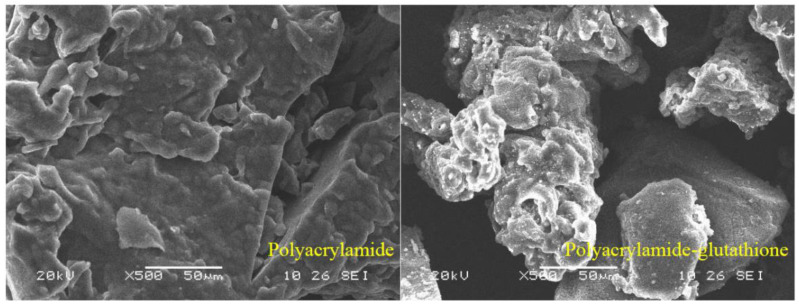
Comparison of before and after polyacrylamide–glutathione synthesis.

SEM was used to compare the surface morphology of polyacrylamide and polyacrylamide–glutathione. It is evident from Figure 5 that the surface of polyacrylamide–glutathione is rougher and has more folds and pores than the surface of polyacrylamide. In addition to a dense pore structure, they exhibit a certain spatial reticulation. Polyacrylamide–glutathione has a strong capacity for adsorption bridging, sweeping net trapping, and aggregation [44,45] due to its complex surface and mesh structure. This causes a continuous process of cadmium ion adsorption in the coagulation process, resulting in an enhanced coagulation performance.

#### 3.2.3. Analysis of the flocculation mechanism

**Figure 6:**
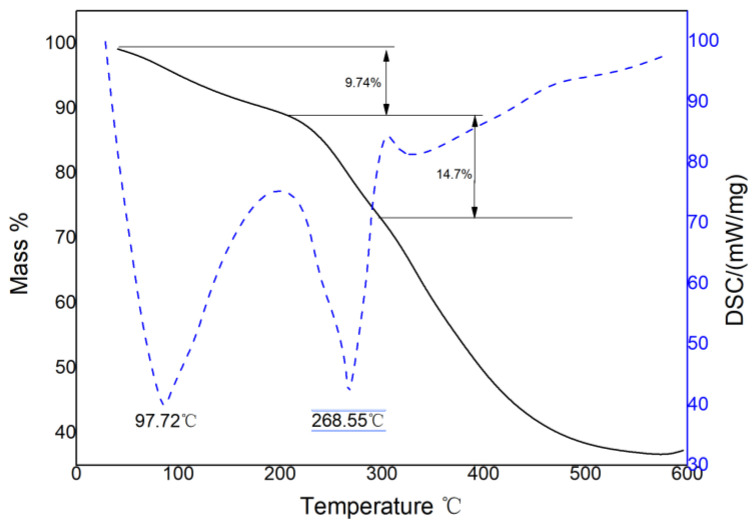
TG-DSC curve.

The polyacrylamide–glutathione sample underwent differential thermal thermogravimetric (TG-DSC) analysis, and the TG-DSC curve depicted in Figure 6 was obtained. The TG curve demonstrated three distinct stages of thermal weight loss. The first stage occurred between 26 and 200 °C with a weight loss of approximately 9.74%, which was attributed to heat-absorbing moisture volatilization of the adsorbed and bound water in the sample, corresponding to the DSC curve with a peak heat absorption temperature of 97.72 °C. In the second stage, between 210~350 °C, the polyacrylamide–glutathione began to decompose, and the molecular structure was damaged by the thermal decomposition of the molecular chain groups (-COOH, -NH_2_, etc.). This led to a weight loss of approximately 14.7%. Finally, the DSC curve displayed a peak in heat absorption at 268.55 °C. With the increasing temperature, the carbon backbone structure of the polyacrylamide–glutathione was gradually broken by thermal decomposition, resulting in weight loss, which corresponds to the heat absorption peak at 335.17 °C on the DSC curve. From the above analysis, it can be seen that polyacrylamide–glutathione has good thermal stability in the range of 26~200 °C and will not decompose.

### 3.3. Performance Testing and Flocculation Mechanism

In this section, batch tests were conducted to investigate the performance of cadmium removal under different conditions. Polyacrylamide–glutathione dosing, water turbidity, pH, and cation presence were the four main conditions. Additionally, the influential processes within each condition were analyzed. The flocculation mechanism was also determined by measuring the zeta potential.

#### 3.3.1. Cadmium Removal Performance Test

**Figure 7:**
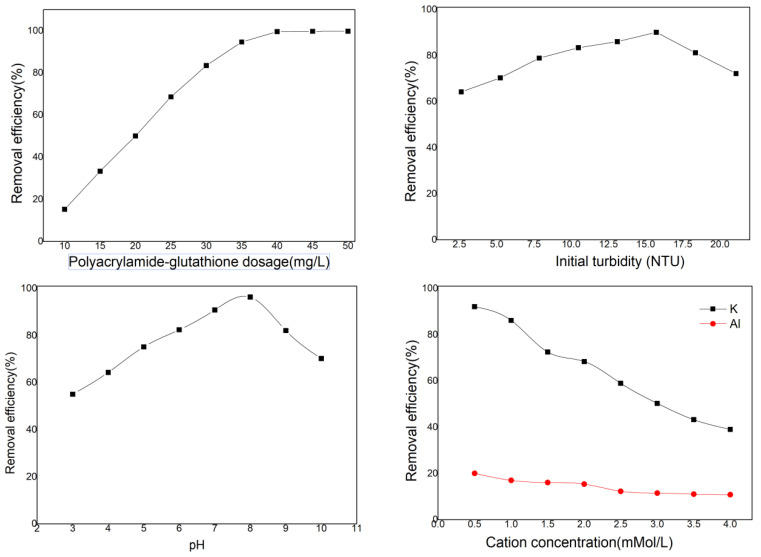
Cadmium removal performance tests.

The measured dissolved concentration of polyacrylamide–glutathione was 10 mg/mL, and the initial concentration of cadmium ions was 20.0 mg L^−1^. The effect of polyacrylamide–glutathione concentration (10–50 mg L^−1^) on cadmium removal was investigated using the experimental coagulation method. Figure 7 demonstrates the outcome. With increasing concentration of polyacrylamide–glutathione, the cadmium removal rate increased gradually and maintained a stable trend. When the concentration was 40 mg L^−1^, the cadmium removal rate reached 99.62%, and as the dosage was increased to 45 and 50 mg L^−1^, it approached the limit at 99.78% and 99.84%, respectively. The optimal dosage of 40.25 mg L^−1^ was determined based on the cost of producing polyacrylamide–glutathione and its effectiveness in removing cadmium.

Figure 7 displays the results of an investigation into the influence of initial turbidity (2.62–15.72 NTU) on the cadmium removal effect. As the initial turbidity increased, the cadmium removal rate first increased and then decreased. In the range of initial turbidity between 2.62 and 15.72 NTU, the cadmium removal rate increased from 64.22 to 90.13%, then, as the initial turbidity further increased, the cadmium removal rate decreased to 72.19%. During the coagulation process, negatively charged kaolin colloidal particles can form small flocs through adsorption and electro-neutralization with positively charged cadmium ions. These flocs co-precipitate to remove cadmium ions from the water, so the moderate turbidity of the water is advantageous for enhancing the cadmium removal effect of polyacrylamide–glutathione. However, an excess of kaolin colloidal particles adsorbed on polyacrylamide−glutathione will obscure the ligand groups and enhance cadmium removal. In light of the initial turbidity and the synergistic effect of polyacrylamide−glutathione on cadmium removal, the optimal initial turbidity was determined to be 13.1 NTU.

As the pH of the initial solution rose, the cadmium removal rate rose and then fell. Under highly acidic or alkaline conditions, the rate of cadmium removal by polyacrylamide–glutathione was less than 80%. When the initial pH of a solution was less than 5, the amino group (-NH_2_) in polyacrylamide–glutathione acquires hydrogen ions, which protonate -NH_2_ to form -NH_3_^+^. The resulting electrostatic repulsion with positively charged cadmium ions is unfavorable for the coordination of the amino group with cadmium ions [46]. whereas the carboxyl group -COO-, which can form coordination bonds with cadmium, is hydrolyzed to -COO- in a highly acidic environment, inhibiting the removal of cadmium. When the solution pH was > 9.0, the cadmium removal rate increased but cannot be attributed to polyacrylamide–glutathione chelation. Under alkaline conditions, most cadmium was precipitated as Cd(OH)_2_, while a small percentage was complexed with amino groups. In this instance, the cadmium removal rate is high, but the polyacrylamide–glutathione was significantly less effective. In light of the synergistic effect of initial pH on cadmium removal, the optimal initial pH range was determined to be between 7.0 and 9.0.

As the cation concentration in the solution system increased, the cadmium removal rate exhibited a continual downward trend. Due to the increasing ligand competition of cations, the cadmium removal rate of polyacrylamide−glutathione decreased. Aluminum ions bind to polyacrylamide–glutathione more strongly than cadmium ions. At lower concentrations, aluminum ions significantly inhibited the formation of complexes between cadmium and polyacrylamide–glutathione ligands, resulting in less than 10% cadmium removal. In the reaction of polyacrylamide–glutathione with positively charged metal ions, the lone pair electrons will delocalize to the empty orbitals of the metal ions and form coordination bonds with them [47], resulting in a charge transfer from the ligand molecule to the metal ions.And then a kind of organic-metal complex was formed. The lone pair of electrons of the donor atoms (N, O, and S) in the polyacrylamide–glutathione ligand molecule can transfer to the empty orbitals of the three metal ions K, Al, and Cd to form complexes. Consequently, potassium and aluminum ions compete with cadmium during coagulation, preventing cadmium removal.

#### 3.3.2. Analysis of the Flocculation Mechanism

In order to comprehend the flocculation mechanism of polyacrylamide–glutathione, the zeta potential changes at different polyacrylamide–glutathione concentration levels (10~80 mg L^−1^) at pH = 4.0, 6.0, and 8.0 were analyzed and determined in this section. The significance of the zeta potential lies in the correlation between its value and the stability of the colloidal dispersion. In addition, the zeta potential quantifies the mutual repulsion or attraction between particles [48].

**Figure 8:**
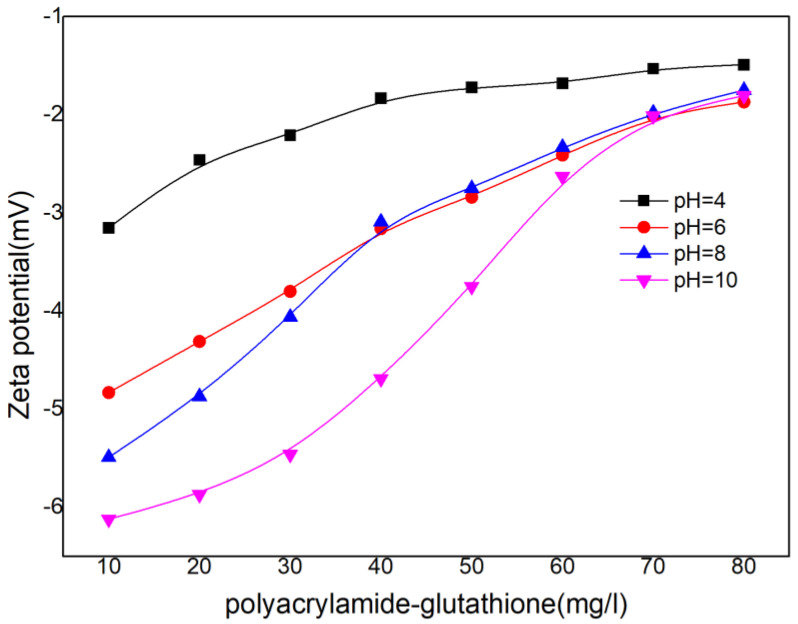
Zeta potential of polyacrylamide–glutathione.

As depicted in Figure 8, the zeta potential changed from negative to zero as the concentration of polyacrylamide–glutathione increased under the three pH conditions. The flocculation process was dominated by adsorption bridging and netting sweeping at pH < 4.0, regardless of the concentration of polyacrylamide–glutathione, and the chelation of ligand groups with cadmium was inhibited. At pH 6.0 to 9.0, the adsorption bridging and netting sweeping effects of polyacrylamide–glutathione were not easily exerted in the low concentration range, and the flocculation effect was primarily attributed to the chelation of -SH on polyacrylamide–glutathione. Chelation of -SH, -COOH, and -NH_2_ ligands on polyacrylamide–glutathione by cadmium ions is primarily responsible for the flocculation effect. Briefly, adsorption bridging and netting sweeping effects were dominant under acidic conditions, and inhibition of chelation increased with increasing solution acidity; as the pH increased, the chelation effect was combined with adsorption bridging and netting sweeping under neutral and alkaline conditions. The adsorption bridging and netting sweeping were primarily determined by the gel’s polyacrylamide properties. According to the DLVO theory [49], the smaller the absolute value of the zeta potential, the weaker the repulsive force between particles, the less stable the dispersion system, and the easier it is for particles to agglomerate, resulting in flocculation [50]. According to the curves and data analysis, the zeta potential of polyacrylamide–glutathione at pH = 10 can reach −6 or even lower at low concentrations. The colloid was more susceptible to destabilization and flocculation under alkaline conditions than under neutral or acidic conditions. This facilitates “adsorption bridging”, “ionic bonding”, and “charge neutralization” in the flocculation process.

## 4. Conclusions

Polyacrylamide-glutathione, a new heavy metal trapping flocculant, was synthesized for the first time in this study to trap the heavy metal cadmium. In addition, the optimal conditions for its synthesis were designed and calculated using response surface methodology. In addition, for the first time, its pertinent characteristics, flocculation mechanism, and application conditions in water bodies were analyzed. The subsequent conclusions were reached:

To optimize the synthetic parameters, response surface synthesis tests were conducted and the significant effects of each parameter were determined in the order of reaction time > polyacrylamide/glutathione mass ratio > pH > formaldehyde dosage. The optimal preparation conditions were a reaction time of 2.692, a mass ratio of 11.17:1 between polyacrylamide and glutathione, and a formaldehyde dosage of 0.49 mL/L. The pH of the reaction system was 3.5. The mean cadmium removal rate of the obtained product was 96.67%, and parallel experiments revealed a relative deviation of 1.05% between the actual value of the cadmium ion removal rate and the theoretical prediction of the model.

The FTIR and EDS results indicated that the polyacrylamide molecular chain contained new sulfhydryl ligand groups. This suggests that glutathione was successfully grafted to polyacrylamide, as opposed to a simple physical mixture of glutathione and polyacrylamide. SEM reveals that the polyacrylamide–glutathione morphology was multi-folded and porous, with a certain reticulation structure, which enhanced the adsorption of cadmium ions in aqueous media and its sweeping and net trapping abilities. The TG-DSC analysis revealed that polyacrylamide–glutathione was thermally stable between 26 and 200 °C and did not decompose.

Experiments investigating the coagulation revealed that polyacrylamide–glutathione was the most effective at removing cadmium ions from a solution with a cadmium concentration of 20.0 mg L^−1^, a dosing amount of 40.25 mg L^−1^, an initial turbidity of 13.10 NTU, and an initial pH of 8.0. In contrast, the presence of two cations, potassium and aluminum, inhibited the cadmium removal process. The primary mechanism of polyacrylamide–glutathione flocculation was chelation, along with adsorption, bridging, netting, and sweeping. Under acidic conditions, adsorption, bridging, and netting are the primary mechanisms and chelation is partially inhibited, while under neutral and alkaline conditions, all three mechanisms operate simultaneously.

## Figures and Tables

**Table 1:** Experimental factors and level controls.

Code	Polyacrylamide/Glutathione Mass Ratio	Reaction Time (h)	Formaldehyde Dosage (mL/L)	pH
−1	1.35	30	0.33	0.5
0	4.05	32.5	0.665	2.25
1	6.75	45	1	4

**Table 2:** Response surface experimental design and result.

	Polyacrylamide/Glutathione Mass Ratio	Reaction Time (h)	Formaldehyde Dosage (mL/L)	pH	Extraction Rate of Cadmium (%)
1	3	0.5	5	1	82.16
2	15	0.5	5	1	84.36
3	3	4	5	1	89.55
4	15	4	5	1	96.31
5	9	2.25	3	0.4	84.26
6	9	2.25	7	0.4	91.02
7	9	2.25	3	1.6	93.5
8	9	2.25	7	1.6	96.55
9	3	2.25	5	0.4	82.21
10	15	2.25	5	0.4	91.57
11	3	2.25	5	1.6	87.62
12	15	2.25	5	1.6	98.07
13	9	0.5	3	1	92.58
14	9	4	3	1	92.33
15	9	0.5	7	1	82.17
16	9	4	7	1	96.58
17	3	2.25	3	1	84.22
18	15	2.25	3	1	90.27
19	3	2.25	7	1	89.01
20	15	2.25	7	1	91.07
21	9	0.5	5	0.4	85.94
22	9	4	5	0.4	93.4
23	9	0.5	5	1.6	85.22
24	9	4	5	1.6	97.67
25	9	2.25	5	1	87.66
26	9	2.25	5	1	94.22
27	9	2.25	5	1	95.03
28	9	2.25	5	1	96.57
29	9	2.25	5	1	96.34

**Table 3:** Comparison of Model Fitting.

Source	Std. Dev.	R^2^	R^2^_Adj_	R^2^_Pre_	
Linear	0.0005	0.5178	0.4731	0.3663	Suggested
2FI	0.4283	0.5139	0.4793	0.2249	
Quadratic	0.0431	0.7643	0.6541	0.2930	Suggested
Cubic	0.5583	0.7844	0.6368	−0.3889	Aliased

**Table 4:** Analysis of variance of response surface quadratic regression model.

Source	Sum of Squares	df	Mean Square	F-value	*p*-value	
Model	640.52	14	45.75	4.78	0.0030	significant
A-P/G ratio	146.16	1	146.16	15.27	0.0016	
B-time	195.29	1	195.29	20.41	0.0005	
C-pH	7.11	1	7.11	0.74	0.4031	
D-formaldehyde	76.15	1	76.15	7.96	0.0136	
AB	22.85	1	22.85	2.39	0.1446	
AC	3.98	1	3.98	0.42	0.5294	
AD	0.30	1	0.30	0.031	0.8627	
BC	53.73	1	53.73	5.62	0.0327	
BD	6.23	1	6.23	0.65	0.4334	
CD	3.44	1	3.44	0.36	0.5583	
A2	103.67	1	103.67	10.83	0.0054	
B2	41.08	1	41.08	4.29	0.0572	
C2	9.82	1	9.82	1.03	0.3283	
D2	4.12	1	4.12	0.43	0.5226	
Residual	133.96	14	9.57			
Lack of Fit	80.58	10	8.06	0.60	0.7643	not significant
Pure Error	53.38	4	13.34			
Cor Total	774.48	28				

**Table 5:** Optimal conditions screening results.

No.	P/G Ratio	Time (h)	pH	Formaldehyde Dosage (mL/L)	Predicted Removal Rate %	Actual Removal Rate	
1	11.170	2.692	4.614	0.419	97.716	96.611	Selected
2	15.000	2.250	5.000	1.600	95.451	95.172	
3	9.000	4.000	7.000	1.000	98.686	97.335	

## Data Availability

Data is contained within the article. More data about this work are available on request from the corresponding author.
